# Proteomic Biomarkers Associated with *Streptococcus agalactiae* Invasive Genogroups

**DOI:** 10.1371/journal.pone.0054393

**Published:** 2013-01-23

**Authors:** Philippe Lanotte, Marylise Perivier, Eve Haguenoer, Laurent Mereghetti, Christophe Burucoa, Stéphane Claverol, Christo Atanassov

**Affiliations:** 1 Equipe “Bactéries et risque materno-fœtal”, UMR 1282 ISP, Université François Rabelais de Tours, Tours, France; 2 Equipe “Bactéries et risque materno-fœtal”, UMR 1282 ISP, INRA, Nouzilly, France; 3 Service de Bactériologie et de Virologie, CHRU de Tours, Tours, France; 4 Service de Bactériologie-Hygiène, CHU de Poitiers, Poitiers, France; 5 Equipe d'accueil 4331 “Laboratoire Inflammation, Tissus Epithéliaux et Cytokines”, Université de Poitiers, Poitiers, France; 6 Pôle Protéomique - Centre de Génomique Fonctionnelle, Université Victor Segalen - Bordeaux 2, Bordeaux, France; Centers for Disease Control & Prevention, United States of America

## Abstract

Group B streptococcus (GBS, *Streptococcus agalactiae*) is a leading cause of meningitis and sepsis in newborns and an etiological agent of meningitis, endocarditis, osteoarticular and soft tissue infections in adults. GBS isolates are routinely clustered in serotypes and in genotypes. At present one GBS sequence type (*i.e.* ST17) is considered to be closely associated with bacterial invasiveness and novel proteomic biomarkers could make a valuable contribution to currently available GBS typing data. For that purpose we analyzed the protein profiles of 170 genotyped GBS isolates by Surface-Enhanced Laser Desorption/Ionization Time-of-Flight Mass Spectrometry (SELDI). Univariate statistical analysis of the SELDI profiles identified four protein biomarkers significantly discriminating ST17 isolates from those of the other sequence types. Two of these biomarkers (MW of 7878 Da and 12200 Da) were overexpressed and the other two (MW of 6258 Da and 10463 Da) were underexpressed in ST17. The four proteins were isolated by mass spectrometry-assisted purification and their tryptic peptides analyzed by LC-MS/MS. They were thereby identified as the small subunit of exodeoxyribonuclease VII, the 50S ribosomal protein L7/L12, a CsbD-like protein and thioredoxin, respectively. In conclusion, we identified four candidate biomarkers of ST17 by SELDI for high-throughput screening. These markers may serve as a basis for further studies on the pathophysiology of GBS infection, and for the development of novel vaccines.

## Introduction

Group B streptococcus (GBS), also referred to as *Streptococcus agalactiae*, is the leading cause of infection in newborns [1]. This bacterial pathogen is also a causative agent of invasive infections in adults such as meningitis, endocarditis, and soft tissue and osteoarticular infections [2]–[4]. Historically, the GBS isolates have been classified into ten different serotypes according to their capsule polysaccharides [5], [6]. Although one of these serotypes, serotype III, is generally associated with late-onset neonatal disease [7], serotyping has turned out to be insufficient to distinguish isolates involved in other clinical outcomes of GBS infection. To improve the diagnostic and prognostic classification of GBS isolates, several molecular biology methods have been developed: multilocus enzyme electrophoresis [8], ribotyping [9], [10], random amplified polymorphism DNA analysis [11], pulsed-field gel electrophoresis [12], and more recently, multilocus sequence typing (MLST) [13]. The application of MLST has contributed to better resolution of GBS isolates and the identification of bacterial genogroups more often associated with invasive infections in newborns [13]. MLST-based classification has been extended by multilocus variable number of tandem repeat analysis (MLVA) [14]. MLVA [13], [14] and other genotyping studies [15], [16] have shown that isolates belonging to one particular genotype cluster, the sequence type 17 (ST17), are associated with more invasive behavior, especially in the late-onset GBS disease in newborns.

A small number of genomic biomarkers of GBS virulence has recently been proposed [13], [17], [18] and several genes, including gbs2018, have been found to be associated with the ST17 genotype cluster [15], [19]. However, these genes are found in no more than 70% of the cases of late-onset meningitis in newborns.

The principal aim of our study was to identify proteomic biomarkers of *S. agalactiae* genogroups that are commonly associated with invasive disease. We used the high-throughput technology Surface-Enhanced Laser Desorption/Ionization Time-of-Flight Mass Spectrometry (SELDI; SELDI ProteinChip) which allows generation and analysis of discriminating protein patterns from hundreds of samples that are tested in a single experiment [20], [21]. Proteomic identification of the statistically significant biomarkers was facilitated by the availability of three complete genomes sequences of *S. agalactiae* strains A909, NEM316, 2603V/R, and the incomplete genome sequences of five strains (18RS21, 515, CJB111, COH1, H36B) (http://cmr.jcvi.org/tigr-scripts/CMR/shared/Genomes.cgi). We analyzed 170 isolates of *S. agalactiae* by SELDI ProteinChip analysis and found four biomarkers which were significantly associated with genogroups defined by MLST, and in particular for isolates from the invasive ST17 and for isolates belonging to closely related genotypes. The purification of these four biomarkers allowed proteomic determination of their primary sequence.

## Materials and Methods

### Bacterial isolates, serotypes and genotyping

The 170 GBS isolates used for SELDI profiling were obtained from cerebro-spinal fluid (CSF) of children with meningitis (n = 54), clinically healthy women with vaginal carriage of this bacterium (n = 54), the respiratory tract of patients with respiratory infections (n = 24), blood cultures from adults patients with endocarditis (n = 15) according to the modified Duke criteria [22], and milk samples from cases of bovine mastitis (n = 23). All GBS isolates were identified by Gram-staining, colony morphology, beta-hemolysis and Lancefield group antigen determination (Slidex Strepto Kit®, bioMérieux, Marcy l'Etoile, France). In addition, the isolates were identified according to capsular serotype with the Pastorex® rapid latex agglutination test (Bio-Rad, Hercules, USA), and by MLST and MLVA [13], [14], [23]. The isolates were representative of the *S. agalactiae* population and belong to the main clonal lineages defined by MLST [13]. MLST was performed previously and data were not available for the 24 isolates from patients with infection of the respiratory tract [14]. Briefly, PCR was used to amplify fragments of about 500 base pairs from seven housekeeping genes (*adhP*, *pheS*, *atr*, *glnA*, *sdhA*, *glcK* and *tkt*) as described by Jones *et al.* [13]. The seven PCR products were purified and sequenced, and an allele number was assigned to each fragment on the basis of its sequence. A sequence type (ST), based on the allelic profile of the seven amplicons, was assigned to each isolate. This previous work made use of the *Streptococcus agalactiae* MLST website (http://pubmlst.org/sagalactiae/) [24]. Based on allelic profile data, a dendrogram was drawn using BioNumerics 6.5 software (Applied Maths, Sint-Martens-Latem, Belgium). An unweighted pair group method using arithmetic averages (UPGMA) was used for cluster analysis. Three reference strains of GBS with completely sequenced genomes (NEM316, 2603 V/R and A909) were used as controls.

### Culture conditions

GBS bacteria were cultured for 24 hours in Todd-Hewitt broth under agitation at 37°C, and the cultures (10 ml) were centrifuged at 3000× *g* for 10 min and at 4°C. The cell pellet was washed in phosphate-buffered saline, pH 7.4 supplemented with PMSF at 0.2 mM final concentration. After centrifugation at 3 000× *g* for 10 min at 4°C, the cell pellet was immediately frozen on dry ice and stored at −80°C.

### Protein extraction

The frozen cell pellets were thawed and resuspended in 1 ml of lysis buffer (16 mM Na_2_HPO_4_, 4 mM NaH_2_PO_4_, 150 mM NaCl, 1% Triton X-100) supplemented with the protease inhibitor cocktail COMPLETE (Roche, ref. 11697498001). The suspension was transferred into a FastPROTEIN BLUE tube and homogenized in a FastPrep apparatus (MP Biomedicals) according to the following protocol: six cycles (40 sec each) at power setting 6, with cooling of the tubes on ice for 5 min between each cycle. After centrifugation at 15 000× *g* for 15 minutes, the supernatants of each sample were divided into several aliquots and stored at −80°C.

### ProteinChip array processing

Two types of ProteinChip ion-exchange arrays, Q10 and CM10, were assembled into a 96-well bioprocessor (Bio-Rad) and preactivated for 30 min with their respective buffers (100 mM Tris-HCl, pH 9.0 or 100 mM sodium acetate, pH 4.0). In the next step, 180 µl of the respective binding buffer for the array was mixed with 20 µl of the protein extract (previously adjusted to a final protein concentration of 0.5 mg/ml in all samples), and incubated for 60 min. All protein samples were tested in triplicate. After two washes with the binding buffers and one quick rinse with HPLC grade water, the spots were loaded twice with 1 µl of a saturated solution of sinapinic acid dissolved in 50% ACN (acetonitrile)/0.5% TFA (trifluoroacetic acid)(v/v). All steps were carried out at room temperature (18–20°C), using the Micromix-5 platform shaker and the robot-pipetting workstation Biomek 3000 (Beckman-Coulter). The arrays were processed in the PCS 4000 ProteinChip Reader (Bio-Rad) which was programmed in a positive ion mode and at ion acceleration potential of 20 kV.

### Spectra processing and statistics using ProteinChip Data Manager 3.0.7 software (Bio-Rad)

After calibration and normalization of all spectra using the total ion current method, clusters of peaks with the same mass were defined at the following settings: S/N (first pass) ≥5, minimum peak threshold: 20%, mass error: 0.3%, S/N (second pass) ≥2. Three types of computer-generated statistics were used for data analysis: the non-parametric Mann-Whitney U test, the Kruskal-Wallis H test, and the method of heat maps/hierarchical clustering.

### Ion-exchange chromatography (IEX)

Protein extracts dissolved in the same lysis buffer as that used for SELDI EDM experiments were dialyzed overnight at 4°C, under agitation against 1000-fold volume of 20 mM Tris-HCl, pH 9.0. The dialyzed samples were fractionated by ion-exchange chromatography using HiTrap Q HP columns (Amersham, ref. 17-1153-01). All IEX steps were carried out at flow rate of 1 ml/min and the column was placed in a column oven at 20°C. A stepwise elution protocol was applied: (i) an initial isocratic step with buffer A (20 mM Tris-HCl, pH 9.5) for 5 min; (ii) a linear gradient between buffer A and buffer B (buffer A with 500 mM NaCl) for 15 minutes: (iii) an isocratic step for 5 minutes with buffer B; (iv) a linear gradient for 10 minutes between buffer B and buffer C (buffer A with 1 M NaCl).

### Reversed Phase - High Pressure Liquid Chromatography (RP-HPLC)

Fractions from the IEX containing the target protein were further subjected to RP-HPLC on Stability columns (CIL Cluzeau, France) of two formats (either C4/300 Å/5 µm/250 mm×4.6 mm or C8/100 Å/5 µm/250 mm×4 mm) using the Perkin-Elmer HPLC system, series 200, and two buffers: buffer A (1% ACN/0.1% TFA) and buffer B (90% ACN/0.1% TFA). Elution from the C4 column involved an initial isocratic step for 10 minutes with buffer A followed by linear gradient between buffer A and B for 20 min. Elution from the C8 column involved an initial isocratic step with buffer A for 10 minutes followed by linear gradient between buffer A and B for the next 5 minutes reaching 75% of buffer B, a second isocratic step for the next 5 minutes with 75% of buffer B, linear gradient between buffer A and B to reach 100% of B for 5 minutes, and a final isocratic step with buffer B for 10 minutes. Other conditions for both C4 and C8 columns: flow rate −1 ml/min; temperature of the column oven −40°C; absorbance −280 nm, fraction size −1 ml.

### Mass spectrometry (MS)-assisted control of protein purity; tricine SDS-PAGE

The fractions from RP-HPLC were concentrated 20-fold in a vacuum centrifuge (miVac, Genevac) to a final volume of ca. 50 µl. Aliquots of 3 µl of the concentrate were spotted on gold arrays (Bio-Rad) and tested in MALDI mode using the SELDI PCS 4000 apparatus with the following acquisition protocol: focus mass 10 000, laser energy 3 000, matrix attenuation 2 500, partition 1/1, 20 shots. The fraction containing the target protein was completely dried in the miVac. It was then reconstituted in tricine SDS sample buffer containing the NuPAGE reducing agent (Invitrogen). This mixture was then divided into two samples that were heated at 40°C for 30 min, and were separated in parallel by 1D Tricine SDS-PAGE using home-cast Tris-tricine gels (18%T/6% C; stacking gel: 2 cm/resolving gel: 16 cm), at a constant 30 V for 1 hour followed by a constant 60 mA for the next 15 hours. Three lanes, one on each side and one in the middle of the gel, were loaded with prestained molecular weight (MW) markers (Fermentas, ref. SM1861). After completion of the electrophoresis, the MW markers served to indicate the approximate position of the target proteins in the unstained gel; thirty 1 mm-thick gel slices covering two adjacent lanes expected to contain the same protein were excised. Each gel slice was further divided into two equal parts each corresponding to one lane. The target protein was extracted from one of these slices by passive elution as described previously [25], and the mass of the passively eluted target protein was confirmed on gold arrays. The proteins in the corresponding second slice were subjected to trypsin digestion and LC-MS/MS microsequencing as described below.

### Trypsin digestion

The protein containing slices were destained in a solution of 25 mM NH_4_HCO_3_/50% ACN and rinsed twice in ultrapure water. They were then shrunk in 100% ACN for 10 min. ACN was removed and the gel pieces were dried at room temperature, covered with the trypsin solution (10 ng/µl, in 40 mM NH_4_HCO_3_ and 10% ACN), rehydrated at 4°C for 10 min, and incubated overnight at 37°C, with rotary shaking. The supernatants were collected, and an extraction solution of ACN/HCOOH/H_2_O (47.5∶5∶47.5, vol∶vol∶vol) was poured onto the gel slices which were agitated for 15 min. This extraction step was repeated twice. The supernatants were pooled, concentrated in a vacuum centrifuge to a final volume of 25 µl, acidified by addition of 1.5 µl of 5% HCOOH, and stored at −20°C.

### NanoLC-MS/MS analysis

Peptide mixtures from each gel slice were analyzed on an Ultimate 3000 Nano LC system (Dionex) coupled to a nanospray LTQ Orbitrap XL mass spectrometer (ThermoFinnigan, San Jose, CA). Ten microliters of peptide digests were loaded onto a 300 µm i.d. ×5 mm C18 PepMapTM trap column (LC Packings), at a flow rate of 30 µl/min. The peptides were eluted from the trap column onto an analytical 75 µm i.d. ×15-cm C18 PepMap column (LC Packings). The mobile phases were a mix of solvent A (0.1% HCOOH/5% ACN) and solvent B (0.1% HCOOH/80% ACN). Elution was performed using a 5–40% linear gradient of solvent B for 35 min. The separation flow rate was set at 200 nl/min. The acquisition in a data-dependent mode alternated between an MS scan survey over an *m/z* range of 300–1700 and five to ten MS/MS scans, with collision-induced dissociation (CID) as activation mode. The MS/MS spectra were acquired using a 2-*m/z* unit ion isolation window and normalized collision energy of 35%. The dynamic exclusion duration was set at 30 sec and monocharged ions were rejected.

### Database search and processing of results

SEQUEST was used through a Bioworks 3.1.1 interface (ThermoFinnigan, San Jose, CA) to search a subset of the NCBI non-redundant database restricted to *Streptococcus agalactiae* entries. Peak lists were created using extract-msn (BioWorks 3.3.1 Thermo Scientific) with the default settings. Data files in the DTA specific format (DTA stands for the extension “.dat”) were generated from the MS/MS spectra that attained a minimal intensity (n≥100) and a sufficient number of ions (n≥5). The DTA file generation authorized the averaging of several MS/MS spectra corresponding to the same precursor ion with a tolerance of 50 ppm. Spectra from peptides with molecular masses between 600 Da and 4500 Da were retained. The search parameters were as follows: mass accuracy of the monoisotopic peptide precursor set to 10 ppm and that for the peptide fragments was set to 0.5 amu. Only b-ions and y-ions were considered for mass calculation. The oxidation of methionine (+16 Da) was considered as a variable modification. Two missed trypsin cleavages were allowed. Only peptides with Xcorr values higher than 2.0 (double charge), 2.5 (triple charge) and 3.0 (more than three charges) were retained. In all cases, we required the peptide p-value to be lower than 0.001 and the DeltaCn value to be above 0.1. All protein identifications were based on the detection of a minimum of two distinct peptides. Using these parameters, we did not detect any false positives. Shared peptides were only counted for the proteins that had the most matching peptides.

## Results

### Assay on reproducibility

In order to evaluate reproducibility, we first checked to what extent selected cell growth and extraction conditions affected stability of the phenotypic protein profiles obtained with the SELDI ProteinChip. The *S. agalactiae* reference strain NEM316 was cultured independently six times under standard growth conditions, and the bacterial cells harvested were subjected to the same extraction procedure, as described in Materials and Methods. The samples were tested on two types of arrays, CM10 and Q10, using identical spectrum acquisition protocols within the range 3000–20000 Da. The protein patterns of the six cultures were perfectly reproducible with respect to presence of all peaks with S/N>2. The coefficients of variation between the global protein patterns were less than 20% for both arrays, and thus within the acceptable range for the SELDI methodology. The protein extract of isolate NEM316 was used as an additional intra- and inter-array control and was systematically tested in all expression difference mapping (EDM) experiments.

### EDM and candidate biomarker selection

The protein patterns of 170 isolates of GBS and of three reference strains of the same taxon were obtained on two types of ProteinChip arrays, CM10 and Q10. The spectral data obtained were subjected to statistical analysis: univariate p-value tests (Mann-Whitney and Kruskal-Wallis) and multivariate heat maps/hierarchical clustering.

**Figure 1:**
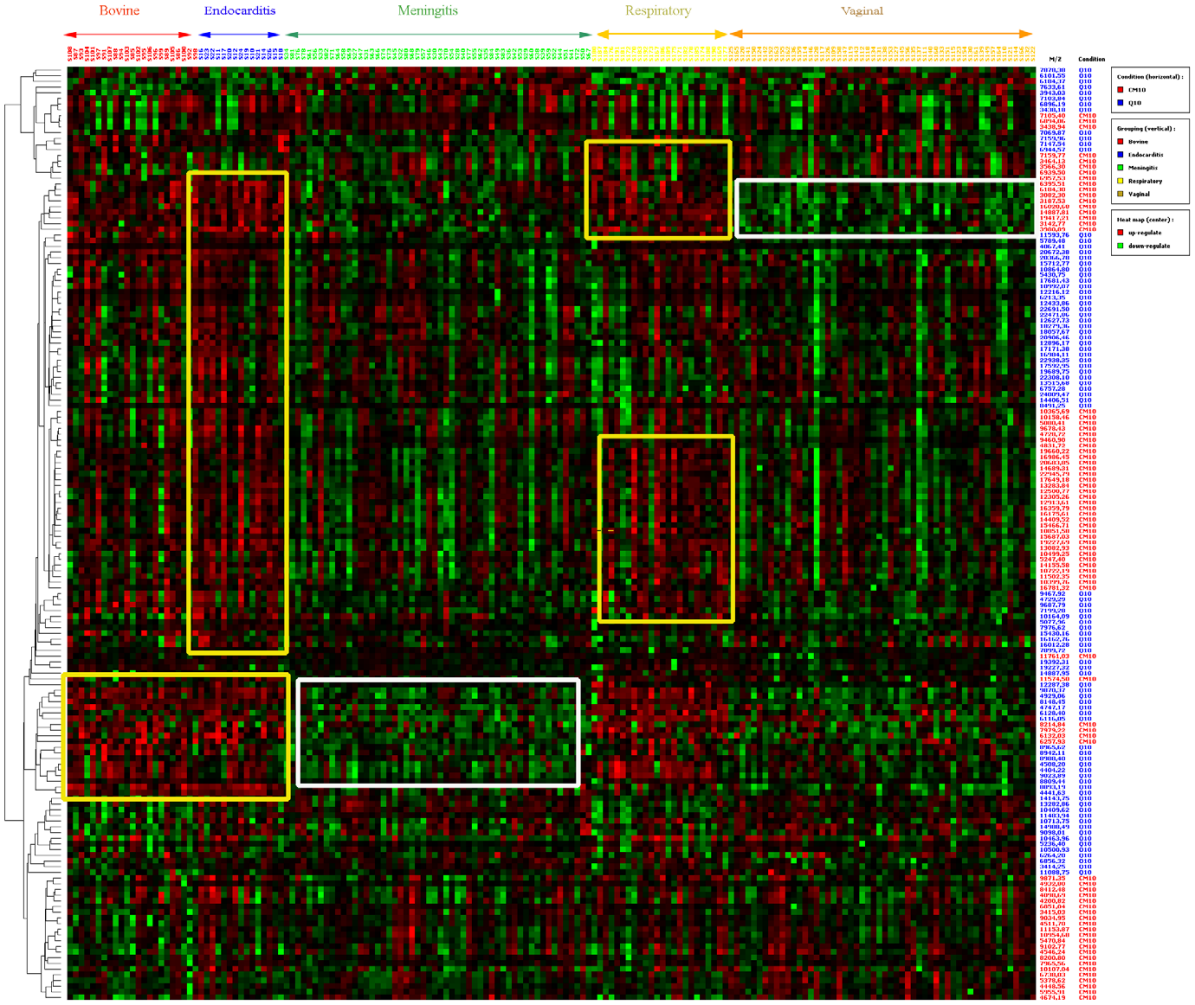
Heatmap/hierarchical analysis of 164 protein clusters of 170 *S. agalactiae* isolates divided into five groups according to origin/clinical outcome: bovine mastitis (n = 23), endocarditis (n = 15), meningitis (n = 54), vaginal carriage (n = 54), and respiratory tract infections (n = 24). The clusters were obtained by combining the results of the ProteinChip array conditions CM10 and Q10. The five isolate group names (upper line, in bold) and the 170 individual isolate names (lower line) are indicated above the image; the protein masses as detected on CM10 (red) and Q10 (blue) ProteinChip arrays are presented.

The initial multivariate analysis (Figure 1) provides a global view of 164 protein biomarkers found in the five GBS groups of isolates (bovine mastitis, respiratory infections, meningitis, endocarditis, vaginal carriage) that are clustered by mass and intensity. Some combinations of biomarkers (arbitrarily grouped into the yellow- and white-bordered rectangles on Figure 1) allow one or two GBS groups to be discriminated from the others, but none of these combinations was completely homogenous in terms of overexpression or underexpression (*i.e.* red or green boxes in the respective rectangles). Another practical consideration was that the number of candidate biomarkers was too large for parallel purification and identification by an academic laboratory like ours.

In order to limit the number of the candidate biomarkers while retaining the most statistically discriminant for further identification, we analyzed the same data with the non-parametric Kruskal-Wallis H test. This test allows simultaneous comparison of the five GBS groups of isolates. In contrast to the previous heat map analysis, the Kruskal-Wallis H test compares the intensity of single biomarkers between the five GBS groups.

In this way, 20 candidate biomarkers were selected with p<0.01 on the Q10 surface and 26 candidate biomarkers with p<0.01 on the CM10 surface (Table S1). However, none discriminated between all the five groups. Most of these biomarkers were overexpressed in one group of isolates, underexpressed in another, and expressed at an intermediate level in the other groups of isolates. Several candidate biomarker patterns were identified with at least two-fold differences in intensity which is a semi-quantitative mode of measurement for protein expression), *e.g.*: *(i)* overexpressed in the endocarditis isolates and underexpressed in the vaginal carriage isolates: p14884 (CM10), p16015 (CM10), p9860 (Q10) and p8889 (Q10); *(ii)* overexpressed in the bovine mastitis isolates and underexpressed in the vaginal carriage and the meningitis isolates: p6258 (CM10), p5787(Q10), p6946 (Q10), p8144 (Q10), p8941 (Q10), p9762 (Q10); *(iii)* overexpressed in the bovine mastitis isolates and underexpressed in the respiratory infection isolates: p12205 (Q10); *(iv)* overexpressed in the respiratory infection isolates and underexpressed in the meningitis isolates (all detected on Q10): p4744, p4926, p6118, p10464, and p19375; *(v)* overexpressed in the meningitis isolates and underexpressed in the respiratory infection isolates (all detected on Q10): p6100, p6182, p7878, and p12200).

As a third statistical analysis, we subjected the spectral data to the non parametric Mann-Whitney test which can be applied only to two groups of variables. Therefore, we clustered the data into two groups according to their membership of the highly virulent ST17 genotype: ST17 and non ST17, irrespective of the isolate origin. An MLST analysis has been reported describing the membership of ST17 for 146 of the GBS isolates included in this study. These isolates belong to the vaginal carriage, meningitis, endocarditis, and bovine mastitis groups. MLST analysis has not been performed on the 24 GBS isolates from respiratory tract infections, and therefore the SELDI spectral data of this group were excluded from the Mann-Whitney test analysis.

Several biomarkers were found to be significantly overexpressed in the group of ST17 isolates, such as p7878 (Q10), p12200 (Q10), whereas others were overexpressed in the non-ST17 isolates, *i.e.* p6258 (CM10), p11581 (CM10), p4745 (Q10), p7905 (Q10), p6118 (Q10), p10464 (Q10), p6945 (Q10) (Table S2).

**Figure 2:**
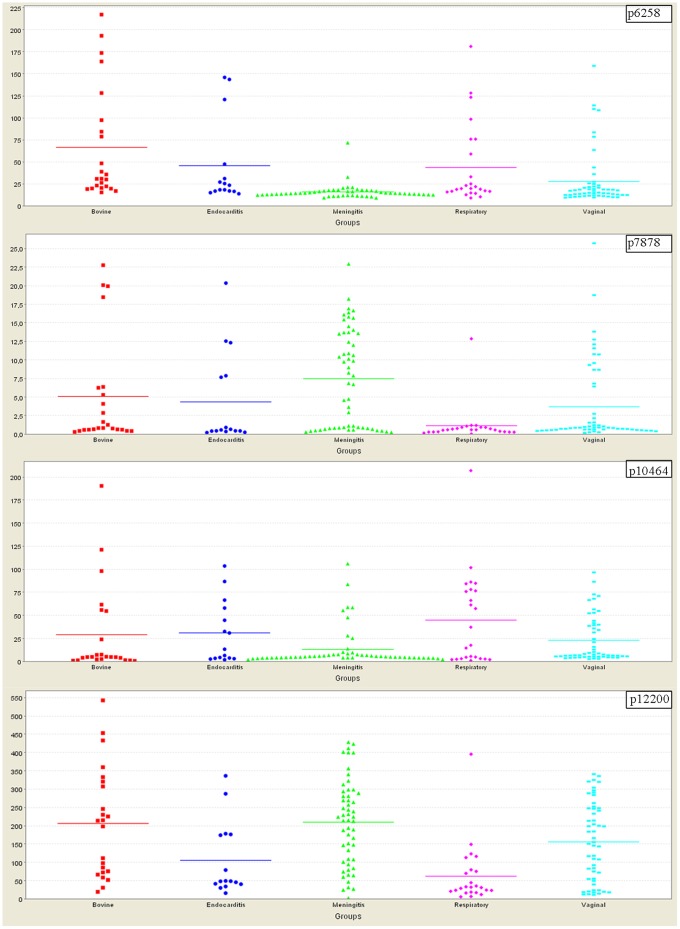
Expression levels of four biomarker proteins produced by *S. agalactiae* isolates clustered into five groups according to origin/clinical outcome. X - axis: bovine mastitis (red), endocarditis (blue), meningitis (green), respiratory infections (magenta), vaginal carriage (cyan). Y- axis: protein abundance expressed as absolute intensity (µA/laser pulse). Each point represents the mean intensity of one sample tested in duplicate. P-values obtained with the Kruskal-Wallis H test: p6258 -<1.10^−6^; p7878 - 0.000059; p10464 - 0.09447; p12200 - 0.000004.

We selected for further structural identification four protein biomarkers, identified both by the Mann-Whitney U test (clustering by ST17 genotype, Table 1), and by the Kruskal-Wallis H test (clustering according to the origin of the *S. agalactiae* isolates; scatter plots on Figure 2). These biomarkers were designated p6258, p7878, p10464 and p12200 where “p” stands for protein and the numbers indicate the mass in Daltons. The biomarker p7878 was of particular interest since it was not only discriminant with a *ca.* 7-fold difference in average intensity between the meningitis isolates and the respiratory infection isolates, but displayed more than a 2-fold difference between the meningitis isolates and the vaginal carriage isolates.

**Table 1:** Biomarkers selected by SELDI profiling of two groups of *S. agalactiae* strains, positive or negative for ST17.

Average mass	p6258	p7878	p10464	p12200
ProteinChip	CM10	Q10	Q10	Q10
Acquisiton protocol*	Protocol 1	Protocol 1	Protocol 1	Protocol 2
p-value (Mann-Whitney test)	0.00089	<1.10^−5^	0.00074	0.0001
Area under the curve (ST17 group selected as positive)	0.33	0.89	0.32	0.71
Number of strains with detectable protein/All tested strains of the ST17 group**	13/47	42/47	2/47	41/47
Mean intensity - ST17 (µA/laser pulse)	15.1	11.3	7.8	229.9
Number of strains with detectable protein/All tested strains of the non ST17 group**	55/99	16/99	43/99	45/99
Mean intensity - non ST17 (µA/laser pulse)	39.4	2.7	29.1	137.1
Mean intensity - ST17/Mean intensity - non ST 17	38.2%	418.5%	26.8%	167.7%

* Acquisiton protocol:

Protocol 1: Focus Mass 10 000, Laser Energy 2 500, Matrix Attenuation 2 500 ; 20 shots ;

Partition 1/4.

Protocol 2 : Focus Mass 15 000, Laser Energy 3 000, Matrix Attenuation 5 000; 20 shots.

Partition 2/4.

** Signal/Noise ratio of detected peaks >2.

**Figure 3:**
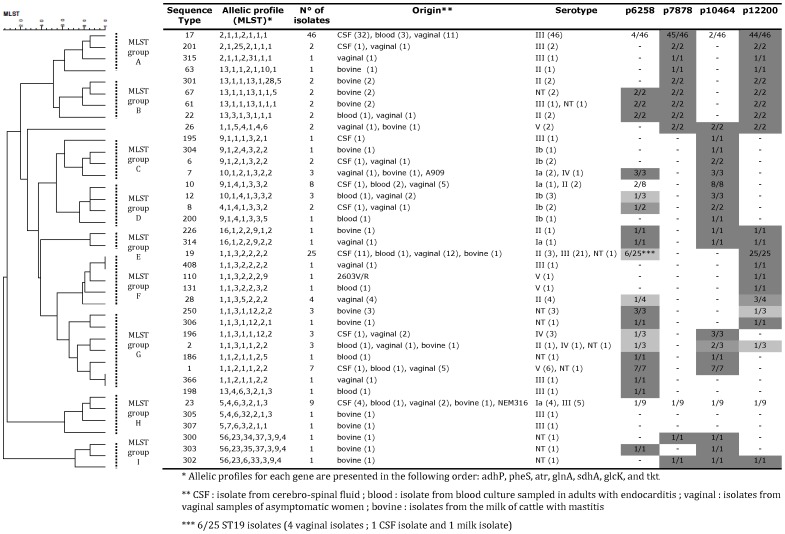
Expression levels of p6258, p7878, p10464 and p12200 in 149 *S. agalactiae* isolates genotyped by multilocus sequence typing (MLST) and represented as a dendrogram showing genetic relationships among the different sequence types (STs). The dendrogram, based on MLST data, was constructed using BioNumerics 6.5 software (Applied Maths, Sint-Martens-Latem, Belgium). Cluster analysis was based on an unweighted pair group method using arithmetic averages (UPGMA). The nine main MLST groups (A to I) are indicated on the right of the dendrogram as vertical dotted lines. The allelic profiles corresponding to each sequence type (ST), the number of isolates belonging to each ST, the origin of the isolate, the serotype and the presence (+) or absence (−) of the biomarkers of interest are reported in detail. Within each ST, the presence of the biomarker is indicated as a ratio between the number of isolates with a detectable corresponding peak in SELDI and the number of all isolates in the ST. A grey scale also illustrates the prevalence of the biomarker (dark grey when the peak corresponding to the biomarker is detected in all isolates; light grey when the peak corresponding to the biomarker is partially detected).

The level of expression of the biomarkers p6258, p7878, p10464 and p12200 in the 146 *S. agalactiae* isolates from the vaginal carriage, meningitis, endocarditis and bovine mastitis groups, as well as from the three reference GBS strains (NEM316, A909 and 2603V/R) was associated with the sequence types (STs) and the MLST groups, as defined by UPGMA analysis (Figure 3). The presence or the absence of these four biomarkers (Figure 3, right part) coincided with the nine MLST groups (A to I) (Figure 3, left part). Thus, the presence of p6258 was associated with isolates in particular STs, *e.g.* ST1 with seven peaks in seven tested isolates (7/7), ST7 (3/3), ST22, ST61, and ST67 (2/2). The biomarker p7878, present in all except one of the 46 isolates belonging to ST17, is also found in all isolates of MLST group A and the closely related MLST group B. The p7878 biomarker is also detected in four isolates which are not clustered in these two MLST groups: ST26 (two isolates), ST300 and ST302 (each with one isolate). The biomarker p10464 is expressed by all of the 14 isolates of ST7, ST10 and ST12, and all 22 isolates belonging to MLST groups C, D and E, as well as in some isolates belonging to MLST group G (particularly ST1 and ST2). However, p10464 was not detected in any of the ST19 isolates of MLST group F (the closest to MLST group G). The biomarker p12200 is present in almost all isolates of MLST groups A and B (54 out of 56), similar to p7878. However, p12200 is also found in isolates of MLST groups E and F, whereas p7878 is not. Finally, the distribution of p10464 and p12200 among sequence types and MLST groups indicates that the presence of these two proteins appears to be mutually exclusive (Figure 3).

### Mass spectrometry (MS)-assisted purification and identification of biomarkers

The four selected biomarker proteins were purified in several steps including ion-exchange chromatography (IEX), reversed phase - high pressure liquid chromatography (RP-HPLC), and one-dimensional sodium dodecyl sulfate-polyacrylamide gel electrophoresis (1D SDS-PAGE). Throughout purification, all fractions obtained were systematically tested for the presence of the target protein by SELDI (the IEX fractions) or matrix-assisted laser desorption/ionization time-of-flight mass spectrometry (MALDI) (the RP-HPLC fractions and those of passive elution from the 1D SDS-PAGE gel slices). The rationale for this approach of mass spectrometry (MS)-assisted purification is that the first liquid chromatography step of fractionation by IEX is carried out under conditions very similar to those of the SELDI profiling experiment. For example, the biomarker p7878 was initially detected in the SELDI expression difference mapping (EDM) experiments on Q10 anion-exchange chips whose active group is a quaternary ammonium. For the initial IEX purification of p7878, we used an anion exchange resin with very similar chemistry to that of the Q10 SELDI chips (*i.e.* prepacked HiTrap Q columns) and the binding buffer was of the same composition and pH as that used in SELDI profiling (*i.e.* 100 mM Tris-HCl, pH 9). Forty IEX fractions obtained after application of a linear gradient of 0–500 mM NaCl were tested in SELDI for the presence of the target protein (p7878) under the same conditions as those of the initial EDM, *i.e.* the same ProteinChip array type, binding buffer, acquisition protocol with identical laser intensity, focus mass, matrix attenuation, spot partition, number of shots kept, etc. In this way, two or three consecutive fractions containing p7878 were selected. During the next RP-HPLC step, the protein-containing fractions were dissolved in ACN/TFA, which allows their rapid concentration under SpeedVac conditions, as well as quality control tests on reusable GOLD arrays (in MALDI mode), in the same mass spectrometer (a PCS 4000) and with the same acquisition protocol. Finally, the proteins were extracted in organic solvent by passive dilution from the half of any gel slice obtained from the 1D SDS-PAGE step, which allows rapid SpeedVac concentration and quality control by MALDI. Only after this final confirmation both of target protein mass and of the absence of contaminants, the other half of the gel was used for sequencing by LC-MS/MS (liquid chromatography coupled to tandem mass spectrometry). For all mass spectrometry experiments (SELDI and MALDI), the spectra were calibrated using a set of reference proteins of known mass. This resulted in only small differences, less than 0.05%, in the masses observed for the target proteins, for example, the p7878 in all IEX, HPLC and 1D SDS-PAGE fractions displayed mass variations of no more than ±3 Da.

The masses of the proteins identified by LC-MS/MS were compared *in silico* to those of peptides in the proteome database for the three complete genomes sequences of *S. agalactiae* strains A909, NEM316, 2603V/R, and the incomplete genome sequences of five strains (18RS21, 515, CJB111, COH1, H36B).

Table 2 shows that the criteria for successful identification (number of different peptides, sequence coverage) are largely fulfilled, and summarizes selected characteristics of the four biomarkers, which were identified as CsbD-like protein (p6258), the small subunit of exodeoxyribonuclease VII (exoDNase VII) (p7878), thioredoxin (p10464), and the L7/L12 subunit of ribosomal protein 50S or RpL7/L12 (p12200).

**Table 2:** Critical parameters of the purified and sequenced protein biomarkers* of *S. agalactiae*.

Biomarker	p6258	p7878	p10464	p12200
Theoretical mass deduced after LC MS/MS** (Average mass found in SELDI)	7064.72 Da (6257.93 Da)	8001.98 Da (7878.38 Da)	11702.91 Da (10 463.96 Da)	12351.57 Da (12 200.33 Da)
Reference	CsbD-like superfamily gi|77413040|ref|ZP_00789242.1| *S. agalactiae* 515^1^	Exodeoxyribo-nuclease VII small subunit gi|77409335|ref|ZP_00786038.1| S. *agalactiae COH1* 2	Thioredoxin gi|25011801|ref|NP_736196.1| *S. agalactiae* NEM316^3^	50S ribosomal protein L7/L12 gi|76787462|ref|YP_329944.1| *S. agalactiae* A909^4^
Aminoacids (AA)	65 AA	71 AA	104 AA	117 AA
Theoretical pI***	pI 7.9	pI 4.26	pI 4.39	pI 4.53
Signal peptide (SP)****	NC	NC	SP 1-33	SP 1-42
Number of MS/MS Scans	8	7	101	35
Number of Different Peptides	4	4	5	9
Sequence Coverage	40.0%	78.9%	58.7%	73.6%

* Whole sequences. The regions of the identified peptides are in bold.

1 MSEEKFDAKVDKVSGSVKESVG**KLTGDKEVESEGKVDKL**KGHA**KEKLADIKDTIKG**ASESFKKKD.

2 MSDK**KTFEENLQELETIVSRLETGDVALEDAIAEFQK**GMLISKELQK**TLKEAEETLVKV**MQADGTEVEMDT.

3 MALEVTDATFVEETKEGLVLIDFWATWCGPC**RMQAPILEQLSQEIDEDELKILKMDVDENPETARQFGIMSIPTLMFKK**DGEVVKQVAGVHTKDQLKAIIAELS.

4 MALNIENIIAEIK**EATILELNDLVKAIEEEFGVTAAAPVAAAA**S**GEAAAAKDSFDVELTAAGDKK**VGVIKVVREIGLKEAK**AIVDNAPSVIKEGASEAEANEIKEK**LEAAGASVTLK.

** Using Mascot and ProFound (Expasy);

*** ProtParam (Expasy);

**** Signal IP 4.0, Gram+ predictions (Expasy) ;

NC : Non Computed by Signal IP 4.0, Gram+ predictions.

The BLAST (Basic Local Alignment Search Tool) results of the first 100 sequences with closest homology/identity (Table 3) show that, with the exception of the CsbD-like protein, the three proteins we identified as biomarkers in *S. agalactiae* are highly conserved and share homology or identity with proteins in other streptococci. Comparison between the proteins in beta-hemolytic *S. agalactiae* and *S. pyogenes* reveals full sequence identity for thioredoxin, 80% maximum identity for the small subunit of exodeoxyribonuclease VII, and 77% maximum identity for the ribosomal protein L7/L12. These three proteins in *S. agalactiae* are most similar (72–83% identity) to those in alpha-hemolytic streptococci (*S. pneumoniae, S. mutans, S. sanguinis*) and enterococci (*E. faecium*). The maximum identity of these proteins between the taxon *S. bovis* and other taxa of streptococci is highest for the thioredoxin (83%), and slightly lower for the small subunit of exoDNase VII (81%) and RpL7/L12 (74%). The CsbD-like protein displays roughly 50% maximum identity with its structural homologs in other *Streptococcus* taxa (Table 3).

**Table 3:** BLAST comparison of the four identifiedprotein biomarkers of *S. agalactiae* to other taxa.

	Maximum identity of identified proteins (%)/Accession number*			
	p6258	p7878	p10464	p12200
Taxon	CsbD-like superfamily	Exodeoxyribo-nuclease VII, small subunit	Thioredoxin	Ribosomal protein L7/L12
*S. agalactiae*	**100%** ZP_08650510.1*,NP_735647.1, YP_329839.1, ZP_00788703.1, ZP_00789242.1, Q8DZG5.1|Y1136_STRA5	**100%** ZP_08651053.1**gb|EAO75233.1) **99%** YP_329230.1, NP_735007.1, ZP_00781258.1, ZP_00788696.1, sp|P67464.1|EX7S_STRA5, ZP_00790175.1	**100%** NP_736196.1**, ZP_00788465.1, YP_002286401.1, ZP_00786146.1, ZP_00789866.1, ZP_00780643.1, ZP_00783054.1, gb|EFV97976.1	**94%** Q8E4M7.1**, YP_329944.1, ZP_00783753.1, ZP_00790846.1 **93%** ZP_00780216.1, ZP_00786275.1, ZP_08650414.1, Q8DZ21.1
*S. pyogenes*	**52%** YP_600627.1	**80%** gb|EAO75233.1	**100%** YP_599235.1	**77%** YP_600464.1
*S. pneumoniae*	**NF**	**74%** NP_358681.1	**79%** gb|EHE02399.1	**78%** CBW36831.1
*S. mutans*	**42%** gb|AAN59280.1|AE014994_10	**73%** YP_003485314.1	**83%** YP_003484192.1	**80%** ZP_08049965.1
*S. sanguinis*	NF	**73%** ZP_08087688.1	**79%** ZP_06612638.1	**77%** ZP_09123485.1
*S. bovis*	**48%** dbj|BAK30866.1	**81%** YP_004287487.1	**83%** gb|EFM26617.1|	**74%** YP_004559300.1
*Enterococcus faecium*	**NF**	**NF**	**NF**	**72%** gb|EFF31024.1

* Accession number on http://www.ncbi.nlm.nih.gov/protein/.

** Sequence identified by LC/MS-MS and used in BLAST to generate the list of the first 100 sequences with closest homology or identity.

**NF:** Absent from the list of the first 100 sequences compared by BLAST.

Software used: BLASTP 2.2.26+ (server - NCBI). Reference: Altschul SF, Madden TL, Schäffer AA, Zhang J, Zhang Z, et al. (1997) Nucleic Acids Res. 25: 3389–3402.

## Discussion

We studied a large selection of 170 representative isolates of *S. agalactiae* to identify proteomic biomarkers that could better characterize their MLST genotypes [14]. Moreover, isolates belonging to the main STs have been found to be associated with particular clinical manifestations: isolates belonging to ST1, ST10 and some genotypically similar STs are often associated with infections in adults [26]; isolates from ST23, ST19 and other closely related STs are associated with vaginal carriage and early infections in newborns [27], [28]; and isolates from ST17 and genotypically related isolates are associated with the late-onset infections in newborns [13], [29]. Although it is tempting to associate these proteomic biomarkers with the different STs and MLST groups of *S. agalactiae* in the context of pathology, our hypothesis requires further experiments using other methods.

Despite extensive genotyping studies, relatively limited information is available about the proteins responsible for GBS virulence and invasiveness [19], [29], [30], [31]. Mass spectrometry can be used to search for proteins with biological activities, and for bacterial classification. Several techniques are currently in use, including MALDI-TOF-MS, LC-ESI-MS and SELDI-TOF-MS. When applied to complex protein samples (*e.g.* bacterial extracts), MALDI and SELDI experiments detect *ca.* 100 different proteins, and typical LC-ESI-MS experiment may reveal more than 500 proteins. However, LC-ESI-MS is labor intensive, time-consuming and requires multiple replicates [32]. We used SELDI, in which proteins are defined by their *m/z*, and protein abundance was estimated in a semi-quantitative manner. Previous MALDI-based studies classified *S. agalactiae* isolates only according to patterns of protein masses [33], [34]. By contrast, we determined protein abundance as being differently distributed among groups of isolates, and identified the primary sequences of some of these potential biomarkers. The predicted masses of the putative biomarkers are slightly higher than those identified by SELDI (Table 2); this may be due to some low-level fragmentation resulting from either the FastPrep sonication of bacteria or proteolysis in the aqueous buffers used. Also, to maximize the purification yield, we used the *S. agalactiae* isolates with the highest detectable levels of the protein for each of the four biomarkers, but none of these isolates was a reference isolate with a completely sequenced genome. The BLAST data (Table 3) indicate that despite the homology, there may be differences in the primary sequences of the proteins produced by different *S. agalactiae* isolates.

According to the EMBL-EBI database (pfam05532), CsbD is a bacterial protein produced in response to general stress. Its expression in *Bacillus subtilis* is mediated by sigma-B [σ(B)], an alternative sigma factor controlling the general stress regulon [35]. Sigma-B is activated in response to numerous physical stress stimuli and conditions of energy starvation. The exact role of CsbD in the stress response is unclear. In *Escherichia coli*, the putative stress-response protein YjbJ, identified by MALDI-TOF-TOF-MS/MS, is considered to belong to the CsbD family, but no particular function has been assigned to this protein [32]. We found no reports concerning CsbD-like proteins in *S. agalactiae*.

Thioredoxins are a family of small redox-active proteins that undergo reversible oxidation/reduction and help to maintain the redox state of cells. They serve as cofactors for a number of enzymes involved in the detoxification of reactive oxygen or nitrogen species. Thioredoxins serve as a cofactor in many thioredoxin reductase-catalyzed reductions in a manner similar to glutathione in thioltransferase reactions. In bacteria, thioredoxins contribute to various important functions such as DNA synthesis (thioredoxin is a hydrogen donor for ribonucleotide reductase), protein disulfide reduction, prevention of oxidative stress, protein repair by methionine sulfoxide reduction, and assimilation of sulfur by sulfate to sulfite reduction [36]. Eukaryotic thioredoxin may be a secreted growth factor or a chemokine for immune cells, which implies potential applications in cancer therapy [37], [38]. There are structural differences between the bacterial thioredoxin reductases, which have low molecular weights, and their mammalian counterparts, which have high molecular weights. These differences could be exploited for the treatment of infections using inhibitors specific for bacterial thioredoxin reductases [37].

The biomarker protein p12200, over-expressed in ST17 isolates of *S. agalactiae*, was identified as the ribosomal protein L7/L12 (RpL7/L12) that has been extensively studied in other bacterial species. RpL7/L12 is a ribosomal 50S protein found under two forms: RpL7 is the acetylated form of RpL12 [39]. In *E. coli*, two RpL7/L12 molecules dimerize and associate with another ribosomal protein, RpL10, to form a stalk complex interacting with translation factors during protein biosynthesis [40]–[44]. RpL7/L12 was identified with the translation factor EF-Ts in culture supernatants of group A streptococci of the M1 and M3 serotypes, suggesting secretion or specific release [44]. The overexpression of this ribosomal protein in *S. agalactiae* isolates may be associated with modulation of the translation of proteins including virulence factors. RpL7/L12 is believed to be a component of a “divisome” protein complex, together with the translation factors EF-Ts and the glucan-binding protein GbpB, involved in cell wall synthesis and expansion in *E. coli* [45], *Streptococcus suis* [46] and *Streptococcus mutans* [47]. RpL7/L12 is a highly antigenic and immunogenic protein [48]–[52] and its high degree of conservation among bacterial species is the cause of many serological cross-reactions. For example, RpL7/L12 purified from the gastric pathogen *Helicobacter pylori* cross-reacts with serum antibodies from *Helicobacter pylori-*negative patients [53]. Mucosal immunization of mice with RpL7/L12, glyceraldehyde-3-phosphate dehydrogenase and four other protein antigens of *S. pneumoniae* enhances bacterial clearance; this protein is therefore a promising vaccine candidate [54].

The most significantly differentially expressed biomarker identified in our study (more than 4-fold more abundant in the ST17 than other isolates) is the small subunit of exodeoxyribonuclease VII (exoDNase VII). It belongs to the family of exonuclease VII small subunits (Exonuc_VII_S superfamily; NCBI - PRK00977). ExoDNase VII (EC: 3.1.11.6) is composed of one large and four small subunits and catalyzes exonucleolytic cleavage in either 5′->3′ or 3′->5′ direction to yield 5′-phosphomononucleotides. Its main biological function is its contribution to DNA mismatch repair (MMR) (http://www.ncbi.nlm.nih.gov/biosystems/5044). MMR is a highly conserved biological pathway that plays a key role in maintaining genome stability. The *Escherichia coli* MMR pathway has been extensively studied [55]: MMR corrects DNA mismatches generated during DNA replication, thereby preventing mutations from becoming permanent in dividing cells. MMR also suppresses homologous recombination and was recently shown to play a role in DNA damage signaling [55].

In the study by Lartigue et al. [34] some ST17 isolates were distinguished from others according to their MALDI protein spectra. Interestingly, two proteins defined by their mass only (*i.e.* MW of 6258 Da and 7625 Da) seem to be very close to two candidate biomarkers identified in our study: CsbD-like (6258 Da) and exoDNase (7878 Da); this previous report agrees with our findings of exoDNase overexpression in ST17 isolates and CsbD-like protein underexpression in the isolates of other sequence types. However, the conditions of protein extraction and mass spectrometry processing in the two studies are different; Lartigue et al. [34] did not purify or sequence the proteins so the identity of the proteins they describe remains unknown.

In conclusion, we have identified four candidate biomarkers that are differentially associated with different genotypes. These findings seem particularly relevant to ST17 and ST17-related genotypes. The underexpression of thioredoxin and CsbD-like protein in some groups of isolates merits further study. The two other candidate biomarkers, RpL7/L12 and exoDNase, are overexpressed in ST17 isolates. Of the four biomarkers identified is exodeoxyribonuclease VII, an enzyme contributing to maintaining genomic stability and adaptative plasticity; we found it to be more than four times more abundant in isolates of the highly invasive ST17 than other isolates of *S. agalactiae*. Although the literature suggests possible involvement of these proteins in pathological mechanisms of infection by *S. agalactiae*, further studies comparing isogenic mutants in functional studies would allow meaningful insights. Their identification, and the availability of their gene sequences, means that it may be possible to produce them as recombinant proteins. This would allow specific antibodies to be raised against the whole molecules and/or to synthetic peptides mimicking their antigenic regions. Potentially, depending upon the biomarker, these reagents could be used for the development of novel vaccines or protein arrays.

## Supporting Information

Table S1 List of the candidate protein biomarkers found in the five studied GBS groups (bovine mastitis, endocarditis, meningitis, respiratory infections, vaginal carriage). Detection on CM10 and Q10 ProteinChip arrays (acquisition protocol 1); p values calculated by the univariate Kruskal-Wallis H test.
(XLS)

Table S2 List of the candidate protein biomarkers found in GBS isolates divided in two genogroups : ST 17 and non ST17. Detection on CM10 (acquisition protocol 1) and Q10 ProteinChip arrays (acquisition protocols 1 and 2); p values calculated by the univariate Mann-Whitney U test.
(XLS)

## References

[1] Bohnsack JF, Whiting A, Gottschalk M, Dunn DM, Weiss R, et al (2008) Population structure of invasive and colonizing isolates of *Streptococcus agalactiae* from neonates of six U.S. Academic Centers from 1995 to 1999. J Clin Microbiol 46: 1285–1291. 18287314 10.1128/JCM.02105-07 PMC2292926

[2] Edwards MS, Rench MA, Palazzi DL, Baker CJ (2005) Group B streptococcal colonization and serotype-specific immunity in healthy elderly persons. Clin Infect Dis 40: 352–357. 15668856 10.1086/426820

[3] Farley MM (2001) Group B streptococcal disease in nonpregnant adults. Clin Infect Dis 33: 556–561. 11462195 10.1086/322696

[4] Schuchat A (2001) Group B streptococcal disease: from trials and tribulations to triumph and trepidation. Clin Infect Dis 33: 751–756. 11512078 10.1086/322697

[5] Lindahl G, Stålhammar-Carlemalm M, Areschoug T (2005) Surface proteins of *Streptococcus agalactiae* and related proteins in other bacterial pathogens. Clin Microbiol Rev 18: 102–127. 15653821 10.1128/CMR.18.1.102-127.2005 PMC544178

[6] Slotved H, Kong F, Lambertsen L, Sauer S, Gilbert GL (2007) Serotype IX, a proposed new *Streptococcus agalactiae* serotype. J Clin Microbiol 45: 2929–2936. 17634306 10.1128/JCM.00117-07 PMC2045254

[7] Musser JM, Mattingly SJ, Quentin R, Goudeau A, Selander RK (1989) Identification of a high-virulence clone of type III *Streptococcus agalactiae* (group B Streptococcus) causing invasive neonatal disease. Proc Natl Acad Sci USA 86: 4731–4735. 2660146 10.1073/pnas.86.12.4731 PMC287347

[8] Quentin R, Huet H, Wang FS, Geslin P, Goudeau A (1995) Characterization of *Streptococcus agalactiae* isolates by multilocus enzyme genotype and serotype: identification of multiple virulent clone families that cause invasive neonatal disease. J Clin Microbiol 33: 2576–2581. 8567885 10.1128/jcm.33.10.2576-2581.1995 PMC228531

[9] Blumberg HM, Stephens DS, Licitra C, Pigott N, Facklam R, et al (1992) Molecular epidemiology of group B streptococcal infections: use of restriction endonuclease analysis of chromosomal DNA and DNA restriction fragment length polymorphisms of ribosomal RNA genes (ribotyping). J Infect Dis 166: 574–579. 1380050 10.1093/infdis/166.3.574

[10] Chatellier S, Huet H, Kenzi S, Rosenau A, Geslin P, et al (1996) Genetic diversity of rRNA operons of unrelated *Streptococcus agalactiae* isolates isolated from cerebrospinal fluid of neonates suffering from meningitis. J Clin Microbiol 34: 2741–2747. 8897176 10.1128/jcm.34.11.2741-2747.1996 PMC229397

[11] Chatellier S, Ramanantsoa C, Harriau P, Rolland, Rosenau A, et al (1997) Characterization of *Streptococcus agalactiae* isolates by randomly amplified polymorphic DNA analysis. J Clin Microbiol 35: 2573–2579. 9316910 10.1128/jcm.35.10.2573-2579.1997 PMC230013

[12] Rolland K, Marois C, Siquier V, Cattier B, Quentin R (1999) Genetic features of *Streptococcus agalactiae* isolates causing severe neonatal infections, as revealed by pulsed-field gel electrophoresis and hylB gene analysis. J Clin Microbiol 37: 1892–1898. 10325343 10.1128/jcm.37.6.1892-1898.1999 PMC84979

[13] Jones N, Bohnsack JF, Takahashi S, Oliver KA, Chan MS, et al (2003) Multilocus sequence typing system for group B streptococcus. J Clin Microbiol 41: 2530–2536. 12791877 10.1128/JCM.41.6.2530-2536.2003 PMC156480

[14] Haguenoer E, Baty G, Pourcel C, Lartigue MF, Domelier AS, et al (2011) A multilocus variable number of tandem repeat analysis (MLVA) scheme for *Streptococcus agalactiae* genotyping. BMC Microbiol 11: 171. 21794143 10.1186/1471-2180-11-171 PMC3163538

[15] Lamy MC, Dramsi S, Billoet A, Reglier-Poupet H, Tazi A, et al (2006) Rapid detection of the “highly virulent” group B streptococcus ST-17 clone. Microbes Infect 8: 1714–1722. 16822689 10.1016/j.micinf.2006.02.008

[16] Luan SL, Granlund M, Sellin M, Lagergård T, Spratt BG, et al (2005) Multilocus sequence typing of Swedish invasive group B streptococcus isolates indicates a neonatally associated genetic lineage and capsule switching. J Clin Microbiol 43: 3727–3733. 16081902 10.1128/JCM.43.8.3727-3733.2005 PMC1233917

[17] Poyart C, Reglier-Poupet H, Tazi A, Billoet A, Dmytruk N, et al (2008) Invasive Group B streptococcal infections in infants, France. Emerg Infect Dis 14: 1647–1649. 18826837 10.3201/eid1410.080185 PMC2609873

[18] Gutekunst H, Eikmanns BJ, Reinscheid DJ (2003) Analysis of RogB-controlled virulence mechanisms and gene repression in *Streptococcus agalactiae* . Infect Immun 71: 5056–5064. 12933848 10.1128/IAI.71.9.5056-5064.2003 PMC187362

[19] Brochet ME, Couve E, Zouine M, Vallaeys T, Rusniok C, et al (2006) Genomic diversity and evolution within the species *Streptococcus agalactiae* . Microbes Infect 8: 1227–1243. 16529966 10.1016/j.micinf.2005.11.010

[20] Issaq HJ, Veenstra TD, Conrads TP, Felschow D (2002) The SELDI-TOF MS approach to proteomics: protein profiling and biomarker identification. Biochem Biophys Res Commun 292: 587–592. 11922607 10.1006/bbrc.2002.6678

[21] Poon TCW (2007) Opportunities and limitations of SELDI-TOF-MS in biomedical research: practical advices. Expert Rev Proteomics 4: 51–65. 17288515 10.1586/14789450.4.1.51

[22] Li JS, Sexton DJ, Mick N, Nettles R, Fowler VG, et al (2000) Proposed modifications to the Duke criteria for the diagnosis of infective endocarditis. Clin Infect Dis 30: 633–638. 10770721 10.1086/313753

[23] Manning SD, Lacher DW, Davies HD, Foxman B, Whittam TS (2005) DNA polymorphism and molecular subtyping of the capsular gene cluster of group B streptococcus. J Clin Microbiol 43: 6113–6116. 16333106 10.1128/JCM.43.12.6113-6116.2005 PMC1317180

[24] Jolley KA, Chan MS, Maiden MC (2004) mlstdbNet - distributed multi-locus sequence typing (MLST) databases. BMC Bioinformatics 5: 86–93. 15230973 10.1186/1471-2105-5-86 PMC459212

[25] Bernarde C, Khoder G, Lehours P, Burucoa C, Fauchere JL, et al (2009) Proteomic *Helicobacter pylori* biomarkers discriminative of low-grade gastric MALT lymphoma and duodenal ulcer. Proteomics Clin Appl 3: 672–681. 21136979 10.1002/prca.200800158

[26] Salloum M, van der Mee-Marquet N, Domelier AS, Arnault L, Quentin R (2010) Molecular characterization and prophage DNA contents of *Streptococcus agalactiae* isolates isolated from adult skin and osteoarticular infections. J Clin Microbiol 48: 1261–1269. 20181908 10.1128/JCM.01820-09 PMC2849610

[27] Martins ER, Pessanha MA, Ramirez M, Melo-Cristino J (2007) Analysis of Group B streptococcal isolates from infants and pregnant women in Portugal revealing two lineages with enhanced invasiveness. J Clin Microbiol 45: 3224–3229. 17699641 10.1128/JCM.01182-07 PMC2045366

[28] Manning SD, Springman AC, Lehotzky E, Lewis MA, Whittam TS, et al (2009) Multilocus sequence types associated with neonatal Group B streptococcal sepsis and meningitis in Canada. J Clin Microbiol 47: 1143–1148. 19158264 10.1128/JCM.01424-08 PMC2668308

[29] Poyart C, Pellegrini E, Gaillot O, Boumaila C, Baptista M, et al (2001) Contribution of Mn-cofactored superoxide dismutase (SodA) to the virulence of *Streptococcus agalactiae* . Infect Immun 69: 5098–5106. 11447191 10.1128/IAI.69.8.5098-5106.2001 PMC98605

[30] Herbert MA, Beveridge CJE, McCormick D, Aten E, Jones N, et al (2005) Genetic islands of *Streptococcus agalactiae* isolates NEM316 and 2603VR and their presence in other Group B streptococcal isolates. BMC Microbiol 5: 31. 15913462 10.1186/1471-2180-5-31 PMC1175089

[31] Spellerberg B (2000) Pathogenesis of neonatal *Streptococcus agalactiae* infections. Microbes Infect 2: 1733–1742. 11137046 10.1016/s1286-4579(00)01328-9

[32] Fagerquist CK, Garbus BR, Miller WG, Williams KE, Yee E, et al (2010) Rapid identification of protein biomarkers of *Escherichia coli* O157:H7 by matrix-assisted laser desorption ionization-time-of-flight-time-of-flight mass spectrometry and top-down proteomics. Anal Chem 82: 2717–27125. 20232878 10.1021/ac902455d

[33] Lartigue MF, Hery-Arnaud G, Haguenoer E, Domelier AS, Schmit PO, et al (2009) Identification of *Streptococcus agalactiae* isolates from various phylogenetic lineages by matrix-assisted laser desorption ionization-time of flight mass spectrometry. J Clin Microbiol 47: 2284–2287. 19403759 10.1128/JCM.00175-09 PMC2708490

[34] Lartigue MF, Kostrzewa M, Salloum M, Haguenoer E, Hery-Arnaud G, et al (2011) Rapid detection of “highly virulent” Group B *Streptococcus* ST-17 and emerging ST-1 clones by MALDI-TOF mass spectrometry. J Microbiol Methods 86: 262–265. 21663770 10.1016/j.mimet.2011.05.017

[35] Reder A, Höper D, Gerth U, Hecker M (2012) Contributions of individual σB-dependent general stress genes to oxidative stress resistance of *Bacillus subtilis* . J Bacteriol 194: 3601–3610. 22582280 10.1128/JB.00528-12 PMC3393503

[36] Arnér ES, Holmgren A (2000) Physiological functions of thioredoxin and thioredoxin reductase. Eur J Biochem 267: 6102–6109. 11012661 10.1046/j.1432-1327.2000.01701.x

[37] Biaglow JE, Miller RA (2005) The thioredoxin reductase/thioredoxin system: novel redox targets for cancer therapy. Cancer Biol Ther 4: 6–13. 15684606 10.4161/cbt.4.1.1434

[38] Lillig CH, Holmgren A (2007) Thioredoxin and related molecules-from biology to health and disease. Antioxid Redox Signal 9: 25–47. 17115886 10.1089/ars.2007.9.25

[39] Bocharov EV, Gudkov AT, Budovskaya EV, Arseniev AS (1998) Conformational independence of N- and C-domains in ribosomal protein L7/L12 and in the complex with protein L10. FEBS Lett 1998 423: 347–350. 10.1016/s0014-5793(98)00121-5 9515737

[40] Koteliansky VE, Domogatsky SP, Gudkov AT (1978) Dimer state of protein L7/L12 and EF-G-dependent reactions of ribosomes. Europ J Biochem 90: 319–323. 361401 10.1111/j.1432-1033.1978.tb12607.x

[41] Nechifor R, Murataliev M, Wilson KS (2007) Functional interactions between the G' subdomain of bacterial translation factor EF-G and ribosomal protein L7/L12. J Biol Chem 282: 36998–37005. 17932030 10.1074/jbc.M707179200

[42] Gudkov AT (1997) The L7/L12 ribosomal domain of the ribosome: structural and functional studies. FEBS Lett 407: 253–256. 9175862 10.1016/s0014-5793(97)00361-x

[43] Wahl MC, Moller W (2002) Structure and function of the acidic ribosomal stalk proteins. Cur Prot Pept Sci 3: 93–106. 10.2174/1389203023380756 12370014

[44] Lei B, Mackie S, Lukomski S, Musser JM (2000) Identification and immunogenicity of group A *Streptococcus* culture supernatant proteins. Infect Immun 68: 6807–6818. 11083799 10.1128/iai.68.12.6807-6818.2000 PMC97784

[45] Nanninga N (1998) Morphogenesis of *Escherichia coli* . Microbiol Mol Biol Rev 62: 110–129. 9529889 10.1128/mmbr.62.1.110-129.1998 PMC98908

[46] Wu Z, Zhang W, Lu C (2008) Comparative proteome analysis of secreted proteins of *Streptococcus suis* serotype 9 isolates from diseased and healthy pigs. Microb Pathog 45: 159–166. 18554861 10.1016/j.micpath.2008.04.009

[47] Mattos-Graner RO, Porter KA, Smith DJ, Hosogi Y, Duncan MJ (2006) Functional analysis of glucan binding protein B from *Streptococcus mutans* . J Bacteriol 188: 3813–3825. 16707674 10.1128/JB.01845-05 PMC1482924

[48] Oliveira SC, Splitter GA (1996) Immunization of mice with recombinant L7/L12 ribosomal protein confers protection against *Brucella abortus* infection. Vaccine 14: 959–962. 8873388 10.1016/0264-410x(96)00018-7

[49] Kolberg J, Hoiby EA, Lopez R, Sletten K (1997) Monoclonal antibodies against *Streptococcus pneumoniae* detect epitopes on eubacterial ribosomal proteins L7/L12 and on streptococcal elongation factor Ts. Microbiology 143: 55–61. 9025278 10.1099/00221287-143-1-55

[50] Kimmel B, Bosserhoff A, Frank R, Gross R, Goebel W, et al (2000) Identification of immunodominant antigens from *Helicobacter pylori* and evaluation of their reactivities with sera from patients with different gastroduodenal pathologies. Infect Immun 68: 915–920. 10639463 10.1128/iai.68.2.915-920.2000 PMC97222

[51] Ribeiro LA, Azevedo V, Le Loir Y, Oliveira SC, Dieye Y, et al (2002) Production and targeting of the *Brucella abortus* antigen L7/L12 in *Lactococcus lactis*: a first step towards food-grade live vaccines against brucellosis. Appl Environ Microbiol 68: 910–916. 11823235 10.1128/AEM.68.2.910-916.2002 PMC126665

[52] Mini R, Bernardini G, Salzano AM, Renzone G, Scaloni A, et al (2006) Comparative proteomics and immunoproteomics of *Helicobacter pylori* related to different gastric pathologies. J Chromatogr B Analyt Technol Biomed Life Sci 833: 63–79. 10.1016/j.jchromb.2005.12.052 16483854

[53] Voland P, Weeks DL, Vaira D, Prinz C, Sachs G (2002) Specific identification of three low molecular weight membrane-associated antigens of *Helicobacter pylori* . Aliment Pharm Ther 16: 533–544. 10.1046/j.1365-2036.2002.01221.x 11876708

[54] Jomaa M, Kyd JM, Cripps AW (2005) Mucosal immunisation with novel *Streptococcus pneumoniae* protein antigens enhances bacterial clearance in an acute mouse lung infection model. FEMS Immunol Med Microbiol 44: 59–67. 15780579 10.1016/j.femsim.2004.12.001

[55] Li GM (2008) Mechanisms and functions of DNA mismatch repair. Cell Res 18: 85–98. 18157157 10.1038/cr.2007.115

